# Supplementary material for: Unveiling the Bioactive Potential of the Invasive Jellyfish Phyllorhiza punctata Through Integrative Transcriptomic and Proteomic Analyses
Source: Biomolecules. 2025 Aug 4;15(8):1121. doi: 10.3390/biom15081121 (PMC12383608; doi:10.3390/biom15081121)
Supplement: Supplementary file 1 [file biomolecules-15-01121-s001.zip › Suplementary Material/FileS3/PEPFOLD4/XP_065051598_10943/clusters.html]

 PEP-FOLD clusters


Download archive of all models  here   

|  |  |  |
| --- | --- | --- |
| # | file | sOPEP |
| # cluster 1 - Size: 1 |  |  |
|  | XP\_065051598\_10943-model1 | -16.1871 |
| # cluster 2 - Size: 2 |  |  |
|  | XP\_065051598\_10943-model2 | -15.0291 |
|  | XP\_065051598\_10943-model2.1 | -9.34126 |
| # cluster 3 - Size: 1 |  |  |
|  | XP\_065051598\_10943-model3 | -13.6683 |
| # cluster 4 - Size: 1 |  |  |
|  | XP\_065051598\_10943-model4 | -12.4432 |
| # cluster 5 - Size: 2 |  |  |
|  | XP\_065051598\_10943-model5 | -11.6926 |
|  | XP\_065051598\_10943-model5.1 | -9.10452 |
| # cluster 6 - Size: 2 |  |  |
|  | XP\_065051598\_10943-model6 | -11.2256 |
|  | XP\_065051598\_10943-model6.1 | -8.31933 |
| # cluster 7 - Size: 1 |  |  |
|  | XP\_065051598\_10943-model7 | -10.7692 |
| # cluster 8 - Size: 1 |  |  |
|  | XP\_065051598\_10943-model8 | -10.4726 |
| # cluster 9 - Size: 1 |  |  |
|  | XP\_065051598\_10943-model9 | -9.78055 |
| # cluster 10 - Size: 1 |  |  |
|  | XP\_065051598\_10943-model10 | -9.70943 |
| # cluster 11 - Size: 1 |  |  |
|  | XP\_065051598\_10943-model11 | -9.55699 |
| # cluster 12 - Size: 1 |  |  |
|  | XP\_065051598\_10943-model12 | -9.46653 |
| # cluster 13 - Size: 1 |  |  |
|  | XP\_065051598\_10943-model13 | -9.39734 |
| # cluster 14 - Size: 1 |  |  |
|  | XP\_065051598\_10943-model14 | -9.29994 |
| # cluster 15 - Size: 1 |  |  |
|  | XP\_065051598\_10943-model15 | -9.18665 |
| # cluster 16 - Size: 1 |  |  |
|  | XP\_065051598\_10943-model16 | -9.09217 |
| # cluster 17 - Size: 1 |  |  |
|  | XP\_065051598\_10943-model17 | -9.05196 |
| # cluster 18 - Size: 1 |  |  |
|  | XP\_065051598\_10943-model18 | -9.04698 |
| # cluster 19 - Size: 1 |  |  |
|  | XP\_065051598\_10943-model19 | -8.92097 |
| # cluster 20 - Size: 1 |  |  |
|  | XP\_065051598\_10943-model20 | -8.89952 |
| # cluster 21 - Size: 1 |  |  |
|  | XP\_065051598\_10943-model21 | -8.84709 |
| # cluster 22 - Size: 1 |  |  |
|  | XP\_065051598\_10943-model22 | -8.79263 |
| # cluster 23 - Size: 1 |  |  |
|  | XP\_065051598\_10943-model23 | -8.74514 |
| # cluster 24 - Size: 1 |  |  |
|  | XP\_065051598\_10943-model24 | -8.74307 |
| # cluster 25 - Size: 1 |  |  |
|  | XP\_065051598\_10943-model25 | -8.37644 |
| # cluster 26 - Size: 1 |  |  |
|  | XP\_065051598\_10943-model26 | -8.3674 |
| # cluster 27 - Size: 1 |  |  |
|  | XP\_065051598\_10943-model27 | -8.35697 |
| # cluster 28 - Size: 1 |  |  |
|  | XP\_065051598\_10943-model28 | -8.28667 |
| # cluster 29 - Size: 1 |  |  |
|  | XP\_065051598\_10943-model29 | -8.25005 |
| # cluster 30 - Size: 1 |  |  |
|  | XP\_065051598\_10943-model30 | -8.19053 |
| # cluster 31 - Size: 1 |  |  |
|  | XP\_065051598\_10943-model31 | -8.16571 |
| # cluster 32 - Size: 1 |  |  |
|  | XP\_065051598\_10943-model32 | -8.11597 |
| # cluster 33 - Size: 1 |  |  |
|  | XP\_065051598\_10943-model33 | -8.11155 |
| # cluster 34 - Size: 1 |  |  |
|  | XP\_065051598\_10943-model34 | -8.04564 |
| # cluster 35 - Size: 1 |  |  |
|  | XP\_065051598\_10943-model35 | -8.03824 |
| # cluster 36 - Size: 1 |  |  |
|  | XP\_065051598\_10943-model36 | -7.90715 |
| # cluster 37 - Size: 1 |  |  |
|  | XP\_065051598\_10943-model37 | -7.85369 |
| # cluster 38 - Size: 1 |  |  |
|  | XP\_065051598\_10943-model38 | -7.76362 |
| # cluster 39 - Size: 1 |  |  |
|  | XP\_065051598\_10943-model39 | -7.76078 |
| # cluster 40 - Size: 1 |  |  |
|  | XP\_065051598\_10943-model40 | -7.6794 |
| # cluster 41 - Size: 1 |  |  |
|  | XP\_065051598\_10943-model41 | -7.6646 |
| # cluster 42 - Size: 1 |  |  |
|  | XP\_065051598\_10943-model42 | -7.63245 |
| # cluster 43 - Size: 1 |  |  |
|  | XP\_065051598\_10943-model43 | -7.60173 |
| # cluster 44 - Size: 1 |  |  |
|  | XP\_065051598\_10943-model44 | -7.57488 |
| # cluster 45 - Size: 1 |  |  |
|  | XP\_065051598\_10943-model45 | -7.57478 |
| # cluster 46 - Size: 1 |  |  |
|  | XP\_065051598\_10943-model46 | -7.54811 |
| # cluster 47 - Size: 1 |  |  |
|  | XP\_065051598\_10943-model47 | -7.5161 |
| # cluster 48 - Size: 1 |  |  |
|  | XP\_065051598\_10943-model48 | -7.47846 |
| # cluster 49 - Size: 1 |  |  |
|  | XP\_065051598\_10943-model49 | -7.38768 |
| # cluster 50 - Size: 1 |  |  |
|  | XP\_065051598\_10943-model50 | -7.35946 |
| # cluster 51 - Size: 1 |  |  |
|  | XP\_065051598\_10943-model51 | -7.34454 |
| # cluster 52 - Size: 1 |  |  |
|  | XP\_065051598\_10943-model52 | -7.33528 |
| # cluster 53 - Size: 1 |  |  |
|  | XP\_065051598\_10943-model53 | -7.30808 |
| # cluster 54 - Size: 1 |  |  |
|  | XP\_065051598\_10943-model54 | -7.28638 |
| # cluster 55 - Size: 1 |  |  |
|  | XP\_065051598\_10943-model55 | -7.21884 |
| # cluster 56 - Size: 2 |  |  |
|  | XP\_065051598\_10943-model56 | -7.19815 |
|  | XP\_065051598\_10943-model56.1 | -6.68478 |
| # cluster 57 - Size: 1 |  |  |
|  | XP\_065051598\_10943-model57 | -7.19588 |
| # cluster 58 - Size: 1 |  |  |
|  | XP\_065051598\_10943-model58 | -7.18042 |
| # cluster 59 - Size: 1 |  |  |
|  | XP\_065051598\_10943-model59 | -7.08642 |
| # cluster 60 - Size: 1 |  |  |
|  | XP\_065051598\_10943-model60 | -7.07405 |
| # cluster 61 - Size: 1 |  |  |
|  | XP\_065051598\_10943-model61 | -7.05015 |
| # cluster 62 - Size: 1 |  |  |
|  | XP\_065051598\_10943-model62 | -7.04802 |
| # cluster 63 - Size: 1 |  |  |
|  | XP\_065051598\_10943-model63 | -7.03111 |
| # cluster 64 - Size: 1 |  |  |
|  | XP\_065051598\_10943-model64 | -7.02844 |
| # cluster 65 - Size: 2 |  |  |
|  | XP\_065051598\_10943-model65 | -6.98711 |
|  | XP\_065051598\_10943-model65.1 | -6.06438 |
| # cluster 66 - Size: 1 |  |  |
|  | XP\_065051598\_10943-model66 | -6.98251 |
| # cluster 67 - Size: 1 |  |  |
|  | XP\_065051598\_10943-model67 | -6.94861 |
| # cluster 68 - Size: 1 |  |  |
|  | XP\_065051598\_10943-model68 | -6.91797 |
| # cluster 69 - Size: 1 |  |  |
|  | XP\_065051598\_10943-model69 | -6.88939 |
| # cluster 70 - Size: 1 |  |  |
|  | XP\_065051598\_10943-model70 | -6.8778 |
| # cluster 71 - Size: 1 |  |  |
|  | XP\_065051598\_10943-model71 | -6.87172 |
| # cluster 72 - Size: 1 |  |  |
|  | XP\_065051598\_10943-model72 | -6.86554 |
| # cluster 73 - Size: 1 |  |  |
|  | XP\_065051598\_10943-model73 | -6.81495 |
| # cluster 74 - Size: 1 |  |  |
|  | XP\_065051598\_10943-model74 | -6.80205 |
| # cluster 75 - Size: 1 |  |  |
|  | XP\_065051598\_10943-model75 | -6.76475 |
| # cluster 76 - Size: 1 |  |  |
|  | XP\_065051598\_10943-model76 | -6.71924 |
| # cluster 77 - Size: 1 |  |  |
|  | XP\_065051598\_10943-model77 | -6.71486 |
| # cluster 78 - Size: 1 |  |  |
|  | XP\_065051598\_10943-model78 | -6.70724 |
| # cluster 79 - Size: 1 |  |  |
|  | XP\_065051598\_10943-model79 | -6.53406 |
| # cluster 80 - Size: 1 |  |  |
|  | XP\_065051598\_10943-model80 | -6.52489 |
| # cluster 81 - Size: 1 |  |  |
|  | XP\_065051598\_10943-model81 | -6.512 |
| # cluster 82 - Size: 1 |  |  |
|  | XP\_065051598\_10943-model82 | -6.42941 |
| # cluster 83 - Size: 1 |  |  |
|  | XP\_065051598\_10943-model83 | -6.40459 |
| # cluster 84 - Size: 1 |  |  |
|  | XP\_065051598\_10943-model84 | -6.38874 |
| # cluster 85 - Size: 1 |  |  |
|  | XP\_065051598\_10943-model85 | -6.35931 |
| # cluster 86 - Size: 1 |  |  |
|  | XP\_065051598\_10943-model86 | -6.31234 |
| # cluster 87 - Size: 1 |  |  |
|  | XP\_065051598\_10943-model87 | -6.3028 |
| # cluster 88 - Size: 1 |  |  |
|  | XP\_065051598\_10943-model88 | -6.25718 |
| # cluster 89 - Size: 1 |  |  |
|  | XP\_065051598\_10943-model89 | -6.22325 |
| # cluster 90 - Size: 1 |  |  |
|  | XP\_065051598\_10943-model90 | -6.16012 |
| # cluster 91 - Size: 1 |  |  |
|  | XP\_065051598\_10943-model91 | -6.09088 |
| # cluster 92 - Size: 1 |  |  |
|  | XP\_065051598\_10943-model92 | -6.07361 |
| # cluster 93 - Size: 1 |  |  |
|  | XP\_065051598\_10943-model93 | -5.92539 |
| # cluster 94 - Size: 1 |  |  |
|  | XP\_065051598\_10943-model94 | -5.9245 |
| # cluster 95 - Size: 1 |  |  |
|  | XP\_065051598\_10943-model95 | -5.56595 |
|  |  |  |
